# Visualizing bovine leukemia virus (BLV)-infected cells and measuring BLV proviral loads in the milk of BLV seropositive dams

**DOI:** 10.1186/s13567-019-0724-1

**Published:** 2019-11-29

**Authors:** Sonoko Watanuki, Shin-nosuke Takeshima, Liushiqi Borjigin, Hirotaka Sato, Lanlan Bai, Hironobu Murakami, Reiichiro Sato, Hiroshi Ishizaki, Yasunobu Matsumoto, Yoko Aida

**Affiliations:** 1 Viral Infectious Diseases Unit, RIKEN, 2-1 Hirosawa, Wako, Saitama 351-0198 Japan; 2 Laboratory of Global Animal Resource Science, Graduate School of Agricultural and Life Sciences, The University of Tokyo, 1-1-1 Yayoi, Bunkyo-ku, Tokyo 113-8657 Japan; 3 Photonics Control Technology Team, RIKEN Center for Advanced Photonics, 2-1 Hirosawa, Wako, Saitama 351-0198 Japan; 4 Department of Food and Nutrition, Jumonji University, Niiza, Saitama 352-8510 Japan; 5 Nakamura Laboratory, Baton Zone Program, RIKEN Cluster for Science, Technology and Innovation Hub, 2-1 Hirosawa, Wako, Saitama 351-0198 Japan; 6 Laboratory of Animal Health II, School of Veterinary Medicine, Azabu University, 1-17-71 Fuchinobe, Chuo-ku, Sagamihara, Kanagawa 252-5201 Japan; 7 Laboratory of Farm Animal Internal Medicine, School of Veterinary Medicine, Azabu University, 1-17-71 Fuchinobe, Chuo-ku, Sagamihara, Kanagawa 252-5201 Japan; 8 Grazing Animal Unit and Nasu Operation Unit, Institute of Livestock and Grassland Science, NARO, 768 Senbonmatsu, Nasushiobara, Tochigi 329-2793 Japan

## Abstract

Bovine leukemia virus (BLV) infects cattle and causes serious problems for the cattle industry, worldwide. Vertical transmission of BLV occurs via in utero infection and ingestion of infected milk and colostrum. The aim of this study was to clarify whether milk is a risk factor in BLV transmission by quantifying proviral loads in milk and visualizing the infectivity of milk. We collected blood and milk from 48 dams (46 BLV seropositive dams and 2 seronegative dams) from seven farms in Japan and detected the BLV provirus in 43 blood samples (89.6%) but only 22 milk samples (45.8%) using BLV-CoCoMo-qPCR-2. Although the proviral loads in the milk tended to be lower, a positive correlation was firstly found between the proviral loads with blood and milk. Furthermore, the infectivity of milk cells with BLV was visualized ex vivo using a luminescence syncytium induction assay (LuSIA) based on CC81-GREMG cells, which form syncytia expressing enhanced green fluorescent protein (EGFP) in response to BLV Tax and Env expressions when co-cultured with BLV-infected cells. Interestingly, in addition to one BLV-infected dam with lymphoma, syncytia with EGFP fluorescence were observed in milk cells from six BLV-infected, but healthy, dams by an improved LuSIA, which was optimized for milk cells. This is the first report demonstrating the infectious capacity of cells in milk from BLV-infected dams by visualization of BLV infection ex vivo. Thus, our results suggest that milk is a potential risk factor for BLV vertical spread through cell to cell transmission.

## Introduction

Bovine leukemia virus (BLV) is the etiological agent for enzootic bovine leukemia (EBL), the most common neoplastic disease of cattle. It belongs to the *Deltaretrovirus* genus of the *Retroviridae* family, which also includes the human T cell leukemia virus types 1 and 2 [1, 2]. Approximately 70% of the BLV-infected cattle show no clinical symptoms, whereas 30% of the infected cattle develop persistent lymphocytosis, which is typified by the polyclonal expression of non-neoplastic CD5^+^ B lymphocyte cells, 2–5% of which form B cell leukemia/lymphoma after a long latency period [1, 2].

Although BLV infects cattle worldwide, effective treatments and vaccines are not available for practical application [2]. In Japan, a recent study showed that 40.9% of dairy cattle are infected with BLV [3]. As such, BLV causes serious problems for the cattle industry. For instance, BLV infection appears to reduce milk production [4], and the annual economic loss to the cattle industry were estimated at $525 million [5]. Therefore, EBL is listed by the World Organization for Animal Health as a problem disease [6, 7]. Under these circumstances, it is necessary to decipher the specific routes of BLV-transmission to prevent the spread of infection and to reduce economic loss [1].

Cell-to-cell transmission is the most efficient route of BLV transmission. The virus is present in circulating peripheral blood lymphocytes of infected cattle, and both horizontal and vertical transmission often occur through infected blood [1]. Vertical transmission occurs via dam-to-calf contact and through in utero infection of the fetus [8], and via the milk and colostrum of naturally-infected cows [9–12]. Indeed, it was previously reported that BLV-infected cells were present in the milk and colostrum of BLV-positive dams, because inoculation of lambs with milk or viable milk cells from 24 dairy cattle naturally infected with BLV resulted in the detection of infectious virus in the milk of 17 cows [13]. Recently, BLV provirus was detected in field samples of milk and colostrum [14–16]. However, the resistance of calves to milk-borne infection can be attributed to virus-neutralizing antibodies, which all calves nursed on BLV-positive dams acquire through the colostrum and retain in their serum for as long as 6 months [17, 18]. In addition, Konishi et al. demonstrated that antibodies in the milk and colostrum of BLV-positive dams could protect against BLV infection in vitro [19]. Therefore, BLV transmission via milk, as compared to contact transmission, occurs at a lower transmission efficiency (around 6–16%) [13, 20–22]. Thus, a critical assessment of these data fails to support the conclusion that BLV transmission occurs via milk. It is therefore essential to evaluate the infectivity of milk and colostrum from BLV-infected cows by performing a detailed in vitro examination of BLV transmission.

The infectivity of several viruses was successfully demonstrated in previous studies [23–26]. For example, the measurement of the infectivity of human immunodeficiency virus (HIV) is based on established reporter cell lines, such as TZM-bl cells, which are stably transfected with a plasmid containing a reporter gene with the HIV long terminal repeat (LTR) in its upstream promoter region that is expressed during HIV replication [25]. Similarly, we developed a luminescence syncytium-induction assay (LuSIA) for assaying the BLV infectivity of CC81-BLU3G cells, which form syncytia expressing enhanced green fluorescent protein (EGFP) when co-cultured with BLV-infected cells [27]. Furthermore, we successfully constructed a new LuSIA protocol that is quantitative and more sensitive than our previous assay, based on CC81-GREMG cells harboring a reporter plasmid containing a mutation in the glucocorticoid-response element in the LTR U3 region of BLV [28]. This new technology enabled us to specifically evaluate the infectivity of peripheral blood mononuclear cells and white blood cells (WBCs) from BLV-infected cows. Unfortunately, no infectivity testing was performed on milk from BLV-positive dams.

Genomic DNA can be extracted from various sources, including whole blood, milk, semen, saliva, nasal secretions, and several organs [14–16, 29–37]. We previously developed a highly specific, accurate, and sensitive method for quantifying proviral loads for both known and novel BLV variants in animals naturally infected with BLV [31, 38–40], based on the use of coordination of common motifs (CoCoMo) primers. Using the BLV-CoCoMo-qPCR-2 assay developed by us, we detected provirus in nasal and saliva samples from cattle with over 14 000 copies/10^5^ cells and 18 000 copies/10^5^ cells in blood samples, respectively [31], suggesting that these cows could be considered to be at a high-risk of BLV transmission via direct contact between infected and uninfected cattle. In addition, it appeared that proviral loads correlated with both BLV infection and disease progression [27, 38, 39]. However, the efficacy of BLV-CoCoMo-qPCR-2 assay has not yet been examined for milk samples from BLV-positive cows.

In recent years, the potential of foodborne spread of BLV from fresh unpasteurized milk and raw beef have increased concern about BLV infection control [14, 41]. The correlation between the blood of dam and the presence of BLV provirus in colostrum has recently been reported [12, 19]; however, the same remains unclear for milk from BLV-positive dam. Therefore, it is important to determine the risk of BLV transmission through milk by quantifying proviral loads in milk and by evaluating the infectivity of milk. This study was undertaken to evaluate the risk of BLV transmission via the milk of BLV-positive dams. We detected the BLV provirus in milk, and visualized and evaluated the BLV infectivity via milk, using the technologies of BLV-CoCoMo-qPCR-2, and LuSIA based on CC81-GREMG cells, previously developed by us. The results of the present study would enable us to establish effective cattle-management policies for the control and eradication of BLV.

## Materials and methods

### Clinical animals and cell lines

Blood and milk samples were obtained from two BLV-negative Holstein–Friesian cattle, 43 BLV-infected Holstein–Friesian cattle without lymphoma, and three BLV-infected Holstein–Friesian cattle with lymphoma from seven farms in Japan (Table 1). CC81-GREMG cells, established from CC81 [28] and FLK-BLV cells, which are persistently infected with BLV, were cultured at 37 °C with 5% CO_2_ in Dulbecco’s modified Eagle’s Medium (DMEM) (Thermo Fisher Scientific, Waltham, MA, USA), supplemented with 10% fetal bovine serum (FBS; Sigma-Aldrich, St. Louis, MO, USA).

**Table 1:** **The BLV-seropositive rates of each farm in this study**

Farm	Cattle no. used in this study	BLV-seropositive rate of the indicated farms^b^, % (+/all)
1^a^	3	Not tested
2	14	15.4 (32/208)
3	12	57.4 (39/68)
4	7	59.7 (37/62)
5	3	54.5 (18/33)
6	7	51.2 (42/82)
7	2	14.9 (31/208)
Total	48	

^a^Farm number 1 included three BLV-infected cattle with lymphoma.

^b^The positive rates for BLV antibodies were determined using an anti-BLV antibody ELISA Kit (JNC, Tokyo, Japan).

### Collection of blood sample, genomic DNA extraction, and isolation of plasma

Ethylenediaminetetraacetic acid (EDTA)-treated whole blood samples (300 µL) were used for genomic DNA extraction with Wizard Genomic DNA Purification Kit (Promega Corporation, Madison, WI, USA), according to the manufacturer’s instructions. Genomic DNA samples were adjusted to 30 ng/µL for use in the BLV-CoCoMo-qPCR-2 assay [38–40]. Separate portions of EDTA-treated whole blood samples were used to separate the plasma.

### Isolation of milk cells, genomic DNA extraction, and LuSIA experiments

To isolate the milk cells, 100 mL milk samples were stored at 4 °C for less than 24 h on the day of sampling, and the cream layer and proteins were removed by performing two sequential centrifugation steps at 4000 × *g* for 3 min in 50 mL sterile tubes. The pellets and remaining supernatants were transferred to new 15-mL sterile tubes and centrifuged at 800 × *g* for 30 min, and the pellets of the milk sample were resuspended in 15 mL phosphate buffered saline (PBS) and washed twice by centrifugation at 620 × *g* for 5 min and at 350 × *g* for *5* min.

DNA was extracted from the milk cells using Wizard Genomic DNA Purification Kit (Promega) with 1.54 mg/mL of dithiothreitol, following the manufacturer’s instructions. The quantity and quality of DNA samples extracted from milk sample was determined based on the A260/280 ratio using a Nanodrop Spectrophotometer ND-1000 (Thermo Fisher Scientific).

The milk cells were also analyzed by LuSIA testing [28]. These cells were resuspended in 1 mL of DMEM, supplemented with 10% FBS and counted using a Neubauer chamber. CC81-GREMG cells were seeded at a density of 5 × 10^4^ cells/well in a 12-well plate (Thermo Fisher Scientific) and cultured at 37 °C for 24 h, after which they were co-cultured with the collected milk cells at densities of 5 × 10^5^, 1 × 10^5^, and 2 × 10^4^ cells/well in culture medium for three days. As a positive control, FLK-BLV cells were co-cultured with CC81-GREMG cells at a density of 5 × 10^4^ cells/well for the same time, as described previously [27]. The culture medium was then replaced with fresh medium at 72 h and the cells were cultured for an additional 24 h. The cells were washed and fixed as described previously [27], and the fluorescent EGFP-positive syncytia in each well were visually counted and scanned by EVOS2 florescence microscopy (Thermo Fisher Scientific) under a 20-fold objective.

To optimize for the milk cells, we developed a modified version of a conventional LuSIA with CC81-GREMG cells, which was optimized for milk cells in terms of (i) the number of CC81-GREMG cells, (ii) the number of milk cells, (iii) the co-culture period, and (iv) the stimulation by pokeweed mitogen (PWM), as shown in Table 3.

### Determination of the BLV proviral loads by BLV-CoCoMo-qPCR-2 assay

The BLV proviral loads in the blood and milk samples were determined with the BLV-CoCoMo-qPCR-2 assay (RIKEN Genesis, Kanagawa, Japan) [38–40] using THUNDERBIRD Probe qPCR Mix (Toyobo, Tokyo, Japan). All amplifications were performed on the Light Cycler^®^ 480 system II (Roche Diagnostics, Mannheim, Germany). The proviral loads were estimated as copy numbers present in 10^5^ white blood cells and milk cells.

### Detection of anti-Env gp51 antibody by enzyme-linked immunosorbent assay (ELISA)

An anti-BLV antibody ELISA Kit (JNC, Tokyo, Japan) was used to detect the anti-Env gp51 antibodies, according to the manufacturer’s instructions.

### Polymerase chain reaction (PCR) amplification and sequencing of BLV *env* gene fragments

Three cattle were randomly chosen for amplification of the BLV *env* gene. The partial BLV *env* genes of blood and milk DNA were amplified by nested PCR, and were purified and sequenced, as described previously [42]. GENETYX (GENETYX Corporation, Tokyo, Japan) and SEQUENCHER software (Hitachi High-technologies Corporation, Tokyo, Japan) were used for editing, alignment, and identification of the nucleotide sequences.

### Statistical analysis

R package version 2.5.2. 2019 was used to calculate *p* value for the significance of the differences between groups. The correlation coefficient (r) was calculated using Excel with the PEARSON function.

## Results

### The quantity and quality of genomic DNA extraction in milk and blood from 48 dams

Blood and milk samples were collected from 48 dams from seven different farms in Japan (Table 1). The rate of detection of BLV antibodies in the plasma of dams from these farms ranged from 14.9 to 59.7%, as determined by conventional serological techniques, such as ELISA (Table 1). Genomic DNAs were extracted from milk cells isolated from 100 mL milk and from whole blood samples of 300 µL.

**Figure 1:**
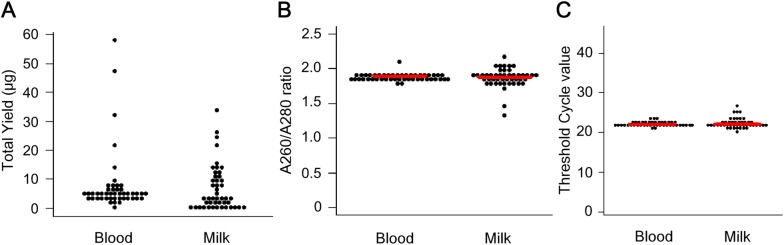
**Scatter diagrams showing the quantity and quality of DNA in blood and milk.** DNA samples were obtained from 2 BLV-negative cattle in Farm 3, 43 BLV-infected cattle without lymphoma in Farms 2–6, and 3 BLV-infected cattle with lymphoma in Farm 1. **A** The average quantities of genomic DNA in blood and milk samples were 7.9 µg and 6.7 µg, respectively, as determined with a NanoDrop spectrophotometer ND-1000. **B** The average A260/A280 ratios of genomic DNA in blood and milk were 1.88 and 1.87, respectively, as determined using a NanoDrop spectrophotometer ND-1000. **C** The threshold cycle values of the blood and milk samples were 22.12 and 22.37, respectively, as indicated with the red bold lines.

The quantity (total yield: A) and quality (A260/A280 ratio: B, threshold value: C) of genomic DNA are shown in Figure 1. The average total DNA yields from 300 µL of blood and 100 mL of milk were 7.9 and 6.7 µg, respectively (Figure 1A). The values of the A260/A280 ratio for genomic DNA extracted from blood and milk were 1.88 and 1.87, respectively (Figure 1B), indicating that DNA extracted from milk and blood had the same quality. To test whether the quality of the extracted DNA was suitable for PCR amplification, and to examine the efficiency of amplification based on the threshold cycle (Ct) value, all the DNA samples were subjected to real-time PCR analysis of the bovine *BoLA*-*DRA* gene, using the BLV-CoCoMo-qPCR-2 assay. *BoLA*-*DRA* was amplified from blood and milk samples from all the cattle tested with average Ct values of 22.12 and 22.37, respectively, indicating that the quality of both the sample types was suitable for PCR amplification (Figure 1C).

### Detection of BLV proviral loads in milk and blood

Next, BLV antibodies were determined in plasma samples from all the 48 cows by BLV antibody ELISA. Forty-six out of 48 cows (95.8%) were positive for the BLV antibodies (Table 2). In parallel, all the samples were analyzed for BLV proviral loads using the BLV-CoCoMo-qPCR-2 assay. Of these, 43 blood DNA samples were positive for the BLV provirus (89.6%), whereas only 22 DNA samples from the milk (45.8%) were BLV provirus-positive. In contrast, no BLV provirus was detected in milk DNA from two dams that were negative for both the BLV antibodies and provirus.

**Table 2:** **Detection of the proviral load in blood and milk samples**

Sample	Age (years)	WBC^a^ (10^2^/µL)	LYMPH^b^ (10^2^/µL)	SCC^c^ (10^6^/mL)	BLV antibodies^e^	Proviral load^f^ (copies/10^5^ cells)	
		Blood		Milk	Plasma	Blood	Milk
BLV-free cows							
K1	2	NT^d^	NT	0.8	–	0	0
K2	2	NT	NT	1.3	–	0	0
BLV-infected cows without lymphoma							
Y2	5	103	53	NT	+	0	0
N6	2	99	38	NT	+	0	0
N7	3	79	23	NT	+	0	0
Y3	7	96	38	NT	+	51	0
G14	6	59	12	NT	+	54	0
S7	9	61	28	NT	+	61	0
S6	4	91	38	NT	+	68	0
K3	3	120	41	1.7	+	112	0
S5	2	80	42	NT	+	361	0
K4	5	69	41	1.6	+	390	0
K5	5	74	34	1.6	+	578	32
G9	2	108	75	NT	+	1457	0
S4	7	77	37	NT	+	3622	0
G11	4	90	16	NT	+	6389	0
N5	7	104	52	2.3	+	10 169	76
G12	6	137	18	NT	+	10 480	0
G10	2	152	26	NT	+	12 097	0
S3	5	105	53	NT	+	17 130	26
G5	4	118	53	NT	+	18 000	32
G8	6	113	54	NT	+	18 793	7
K6	3	78	19	NT	+	19 548	19
S1	7	109	60	NT	+	20 139	0
K7	3	119	68	6.4	+	21 267	0
G4	8	114	43	NT	+	21 284	51
G2	6	85	47	NT	+	21 603	0
G13	3	116	NT	NT	+	22 331	0
G7	3	87	16	NT	+	24 387	0
GDF211	8	82	45	NT	+	24 694	56
G6	6	104	61	NT	+	25 139	70
S2	3	150	94	NT	+	26 002	0
N4	6	93	33	NT	+	27 642	0
Y1	8	72	28	NT	+	34 831	64
GDF349	6	142	83	NT	+	35 859	66
N2	4	158	92	NT	+	35 758	83
N1	3	86	38	NT	+	37 707	159
N3	2	134	89	2.0	+	39 024	0
G3	7	106	45	NT	+	39 237	43
K8	3	175	120	1.2	+	53 985	179
K9	4	214	131	NT	+	58 734	0
G1	5	168	105	9.1	+	65 000	95
K10	5	206	140	1.2	+	72 014	54
K11	7	NT	NT	0.5	+	78 629	129
K12	4	NT	NT	1.0	+	84 479	47
BLV-infected cows with lymphoma							
A1	8	109	NT	4.5	+	4500	6340
A2	6	196	157	2.5	+	65 782	422
A3	5	NT	NT	NT	+	111 564	125

^a^WBC: White blood cell.

^b^LYMPH: lymphocyte.

^c^SCC: somatic cell count.

^d^NT: not tested.

^e^ELISA testing was performed using an anti-BLV ELISA kit (JNC Inc.) targeting BLV gp51. +, positive for anti-BLV antibodies; −, negative for anti-BLV antibodies.

^f^Proviral loads per 10^5^ cells in blood and milk were measured via BLV-CoCoMo-qPCR-2 assay (RIKEN Genesis) [40], using THUNDERBIRD Probe qPCR Mix (Toyobo).

The BLV proviral DNA was detected in both the blood and milk samples from 22 cattle; the proviral loads of blood samples (578–1.1 × 10^5^ copies/10^5^ cells; average, 4.0 × 10^4^ copies/10^5^ cells) tended to be higher than those of milk samples (7–6.3 × 10^3^ copies/10^5^ cells; average, 371.5 copies/10^5^ cells) (Table 2).

### Correlation of BLV proviral loads in milk and blood samples

**Figure 2:**
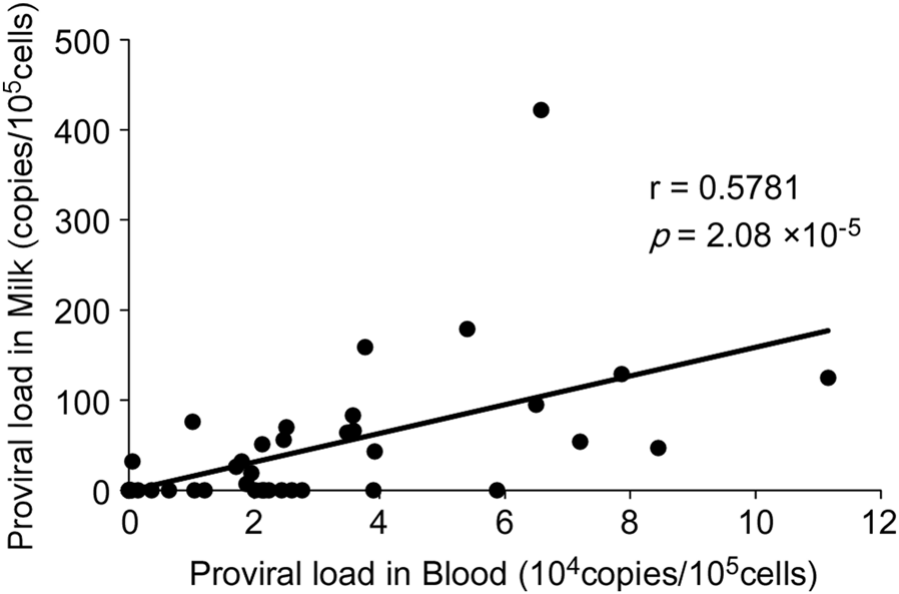
**Correlation between the BLV proviral loads in blood and milk.** The proviral loads in milk and blood samples from 47 cattle in Farms 1–6 were measured by BLV-CoCoMo-qPCR-2 and compared after normalization to the levels found in 10^5^ cells. The bold line represents the approximate curve (*r* = correlation coefficient) and *p* value is indicated.

The proviral load in the peripheral blood appeared to correlate with both the BLV infection and disease progression [27, 38, 39]. Therefore, to examine whether the BLV proviral loads in the peripheral blood samples correlated with those in the milk samples, we constructed a scatter graph using samples from two uninfected and 45 infected cattle (excluding lymphoma cattle A1) and performed a linear-regression analysis. We omitted the lymphoma cattle A1 from this calculation because it had a tumor in the posterior quarter of the mammary gland, and because it exhibited a proviral load that was 15–50-fold higher than that measured in milk from other lymphoma cows, such as A2 and A3. As shown in Figure 2, the correlation coefficient (r) was 0.5781 (*p* = 2.08 × 10^−5^), indicating that a correlation existed between the DNA proviral loads in milk and blood samples from the BLV-infected dams.

In addition, we detected the BLV provirus in milk samples from dams when the proviral loads in blood samples were approximately > 10 000 copies/10^5^ cells (excluding K5) (Table 2).

Our results suggest that these cows may be considered as being at high-risk for BLV transmission via milk from naturally infected dams.

### Comparison of the nucleotide sequences obtained from milk and blood samples

**Figure 3:**
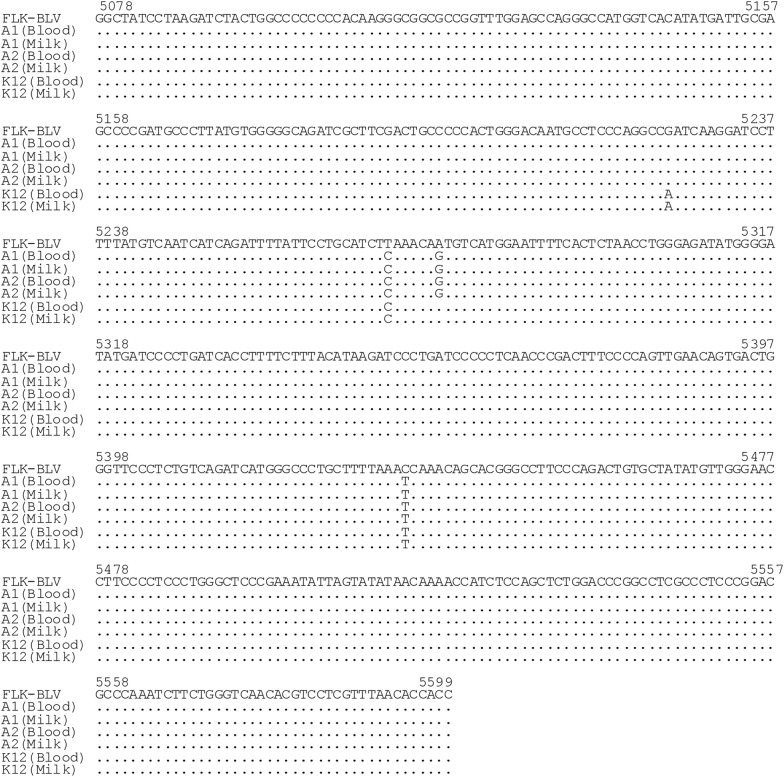
**DNA-sequence alignment of the partial BLV gp51** ***env*** **gene region isolated from blood and milk.** The FLK-BLV sequence (EF600696) is shown as reference at the top of the sequence alignment. DNA fragments derived from blood and milk samples of two BLV-infected cattle with lymphoma (cows A1 and A2) and a BLV-infected cow without lymphoma (cow K12). The nucleotide (nt) sequences were compared with the FLK-BLV of gp51 *env* region (5078 nt–5599 nt). Sequence identity with the FLK-BLV sequence is indicated with dots.

We tried to confirm whether PCR products amplified from milk samples in the BLV-CoCoMo-qPCR-2 assay were derived from BLV, and whether the nucleotide sequences of the products from milk and blood samples were identical in the same animals. The BLV gp51 *env* gene sequences have been detected in BLV seropositive cattle from different global locations and are widely used for molecular characterization and genotyping. Therefore, we determined partial sequences of BLV *env* from milk and blood samples using three different samples (A1, A2, and K12) and aligned these sequences with corresponding sequences from a reference strain, EF600696 (FLK-BLV; Figure 3). The results clearly showed that the partial BLV *env* sequences of the isolates from milk samples from all the three BLV-infected cows were completely identical to those from blood samples of the same animals. The sequencing results showed that the isolates identified in this study belonged to genotype 1 [6].

### Visualization of the BLV-infected cells in milk from BLV-positive dams by conventional LuSIA testing

**Figure 4:**
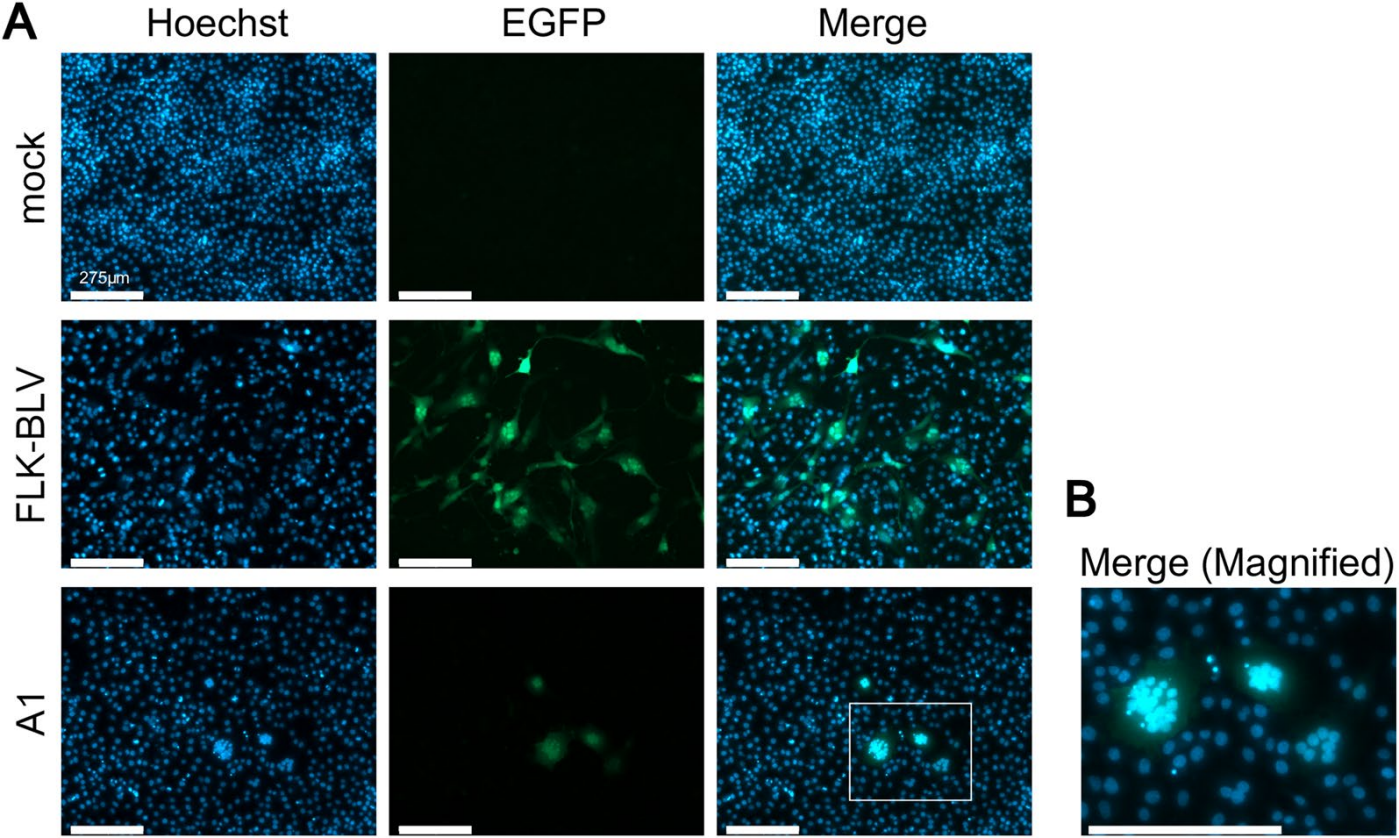
**BLV infectivity of milk cells from a BLV-infected cow with lymphoma (A1) via conventional LuSIA. A** Milk cells were isolated from 100 mL milk sample and were resuspended in 1 mL of DMEM. Then milk cells (1 × 10^5^) were cultured for 4 days with CC81-GREMG cells. Subsequently, the cells were fixed in 3.6% formaldehyde/PBS with Hoechst 33342. The fluorescent syncytia were observed using an EVOS2 fluorescence microscope. FLK-BLV cells, which were productively infected with BLV, were used as the positive control. Mock-treated cells were used as the negative control. **B** Low-magnification image of the white square in subpanel A1. The scale bars (white bars) signify 275 µm.

We also evaluated the BLV infectivity of milk cells. To determine whether LuSIA is useful for assessing the BLV infectivity of milk cells from the BLV-infected dams, we performed a conventional LuSIA by co-cultivating CC81-GREMG cells (5 × 10^4^ cells/well) expressing EGFP with milk cells (5 × 10^5^, 1 × 10^5^, or 2 × 10^4^ cells/well; obtained from 100 mL milk samples) for 4 days (Table 3). Ten BLV provirus-positive milk samples (from cows K5, K6, K8, K10, K11, K12, A1, A2, N1, and N5) and the five milk samples (from cows K1, K2, K3, K4, and K9) that were negative for the provirus were used in the LuSIA experiment. Interestingly, fluorescent syncytia were only observed when CC81-GREMG cells were co-cultured with milk cells from the BLV-positive cow, A1, which had lymphoma (Figure 4). In contrast, no fluorescent syncytia were detected in milk cells isolated from the other cows. In particular, cow A1 had a tumor in the posterior quarter of the mammary gland and showed a proviral load in the milk (6340 copies/10^5^ cells) that was higher than that in the blood (4500 copies/10^5^ cells), as shown in Table 2. Although LuSIA testing with CC81-GREMG cells was useful for visualizing the BLV infectivity of milk cells, it was necessary to optimize the detection conditions for milk cells from other cows.

**Table 3:** **Differences in the conditions used for the conventional and improved LuSIAs with CC81-GREMG cells**

Parameter	Conventional	Improvement
The number of CC81-GREMG cells	5 × 10^4^ cells/well	1 × 10^4^ cells/well
The number of cells in the milk	5 × 10^5^, 1 × 10^5^, and 2 × 10^4^ cells/well	1 × 10^5^ cells/well
Days of co-culture	96 h (4 days)	120 h (5 days)
Activation of B-cells by pokeweed mitogen stimulation	None	1 µg/mL

### Visualization of the BLV-infected cells in milk from BLV-positive dams using an improved LuSIA

**Figure 5:**
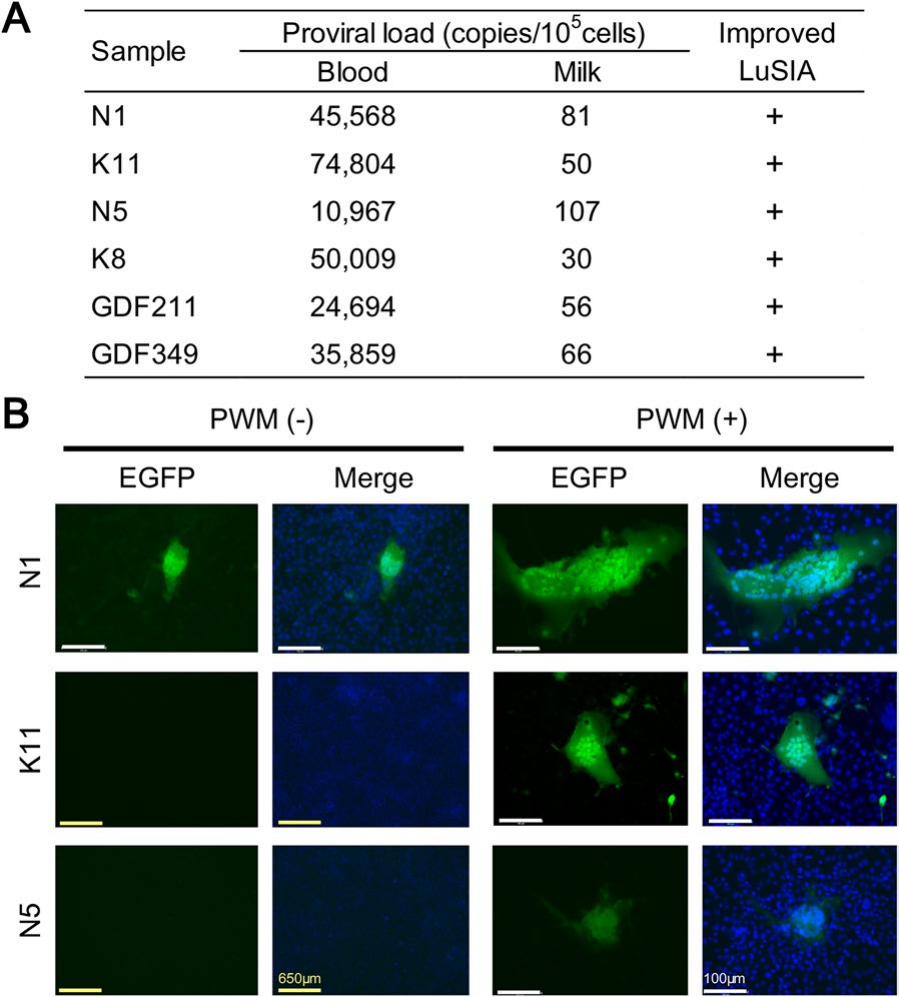
**Infectivity of milk cells from BLV-infected, healthy cows with our improved LuSIA using CC81-GREMG cells. A** The proviral loads in blood and milk, and the improved LuSIA results of milk cells for 6 BLV-infected cattle without lymphoma (cows N1, K11, N5, K8, GDF211 and GDF349). **B** Typical visualization of the infectivity of milk cells from selected cows N1, K11, and N5 among 6 BLV-infected dam tested in this study using our improved LuSIA protocol. The cells remaining in the supernatants were pelleted at 800 × *g* (cows N1 and K11) or 1000×*g* (cow N5) for 30 min. Milk cells (1 × 10^5^) were cultured with 1 × 10^4^ CC81-GREMG cells with (+) or without (−) 1 µg/mL of PWM. After 5 days of co-culturing CC81-GREMG cells with milk cells, the cells were fixed in 3.6% formaldehyde/PBS with Hoechst 33342. Fluorescent syncytia were observed using an EVOS2 fluorescence microscope. +, more than two syncytia were observed in the milk cells. FLK-BLV cells, which were productively infected with BLV, were used as the positive control. The scale bars (white and yellow bar) signify 100 µm or 650 µm.

We next developed a modified version of a conventional LuSIA with CC81-GREMG cells that was optimized for milk cells in terms of (i) the number of CC81-GREMG cells, (ii) the number of milk cells, (iii) the co-culture period, and (iv) PWM stimulation (Table 3). We selected three BLV-infected cows (N5, N1, and K11), which had proviral loads of 76, 159, and 129 copies/10^5^ cells in the milk, respectively (Table 2) and reanalyzed proviral loads in milk (Figure 5A). When the number of CC81-GREMG cells were reduced by 1 × 10^4^ cells/well in assays with 1 × 10^5^, 5 × 10^5^, or 1 × 10^6^ milk cells/well, and increasing the co-culture time to 5 days, fluorescent syncytia were observed in milk cells from two of three cows, namely N1 (1 × 10^5^ cells/well, Figure 5B, left panel) and N5 (5 × 10^5^ cells/well or 1 × 10^6^ cells/well, data not shown), which were not detected using the conventional LuSIA protocol. By contrast, fluorescent syncytia were not observed in N5 (1 × 10^5^ cells/well) and K11 (1 × 10^5^ cells/well) (Figure 5B left panels). Finally, we analyzed the effect of activating the B cells from cows N1, N5, and K11 via PWM stimulation (Figure 5B, right panels). We added 0.2, 1, 10, or 20 µg/mL of PWM to CC81-GREMG cells and co-cultured them with milk cells for 5 days. Fluorescent syncytia were observed in milk cells from all the three cattle when only 1 µg/mL of PWM was added during the co-culture of 1 × 10^4^ CC81-GREMG cells/well and only 1 × 10^5^ milk cells/well among several cell concentrations of milk cells tested (Figure 5B, right panels). Interestingly, the fluorescent syncytia became detectable in milk cells from K11 for the first time, following cellular activation by PWM stimulation. In addition, milk cells from cows N1, N5, and K11 exhibited stronger fluorescence and formed larger and more number of syncytia after PWM stimulation. Thus, as shown in Table 3, the improved LuSIA protocol was optimized using 1 × 10^4^ CC81-GREMG cells/well, 1 × 10^5^ milk cells/well, a 5-day co-culture period, and stimulation with 1 µg/mL of PWM.

Finally, we applied the improved LuSIA to other three BLV-infected cows (K8, GDF211 and GDF349), with proviral loads of 30, 50 and 66 copies/10^5^ cells in the milk, respectively, and successfully observed the fluorescent syncytia in milk cells from the three BLV-infected cows tested (Figure 5A). The present data demonstrate that milk cells from BLV-positive dam have BLV infectivity.

## Discussion

Based on the results of the present study on milk taken from BLV-infected dams, we arrive at two conclusions discussed below. First, the BLV provirus was detected in milk samples from 22 out of 48 cattle using the BLV-CoCoMo-qPCR-2. In addition, the proviral load in milk was positively associated with that in the blood, indicating that the proviral load in the peripheral blood is a useful marker of the proviral load of milk cells. Our results also show that it is difficult to detect BLV in milk as compared to blood samples. Previously, the correlation between the proviral load in the blood of dam and the presence of provirus in the colostrum was investigated [12]. This is the first report demonstrating a positive correlation between the proviral loads in normal milk and blood, in addition to the colostrum, among BLV-infected cattle. Second, the BLV infectivity of milk cells from seven BLV-infected dam was successfully determined ex vivo using a LuSIA based on CC81-GREMG cells. Among the milk samples from 10 cows positive for the BLV provirus, the milk cells from one BLV-infected dam with lymphoma appeared to have infectivity, as determined by the conventional LuSIA. Therefore, we developed a new and highly sensitive LuSIA, which was optimized for milk cells. Six BLV-infected, but healthy, dams without lymphoma appeared to have infectivity, as determined by the improved LuSIA. In particularly, we demonstrated that there is infectious capacity of cells in milk when the proviral loads in milk samples were at least 30 copies/10^5^ cells, as shown in Figure 5. On the other hand, our current data show that there is infectious capacity of cells in blood by the LuSIA when the proviral loads in blood are at least 4 copies/10^5^ cells (Unpublished data). This is the first report indicating the infectious capacity of cells in milk from BLV-infected cows ex vivo. Thus, these results suggest that BLV transmission to cattle might be caused by the consumption of raw milk. However, it was previously reported that antibodies in milk and colostrum of BLV-positive dams appeared to protect against BLV infection in vitro [19]. Therefore, it is essential to evaluate the effect of neutralizing antibody on the infectivity of milk samples by our improved LuSIA.

Our results provide a strong evidence that the BLV proviral load in the milk is positively associated with the proviral load in the peripheral blood. Interestingly, partial sequence analysis of the BLV gp51 *env* gene revealed that the BLV strains in milk samples were identical to those in the matched blood samples from all the three cows analyzed in this study. These results show that, although it is unknown whether peripheral blood or organs maintain BLV proliferation, BLV-infected cells derived from peripheral blood circulate throughout the body and are then distributed in the mammary glands in vivo and, thus, milk includes BLV-infected cells. In addition, the proviral loads in the peripheral blood were reflected in the levels of BLV-infected cells in milk as well as nasal and saliva [31]. Thus, it appears that proviral loads in the peripheral blood represent a useful marker for following the dynamics of BLV-infected cells in vivo. In contrast, a previous study revealed that BLV provirus does not have any correlation with antibodies in plasma and milk samples [15].

This study clearly shows that, although the BLV provirus was detected in milk cells, the frequency of detection (45.8%) was lower than that in blood (89.6%). In addition, the proviral loads in milk tended to be lower than those in the blood from the same animals. Likewise, previous reports showed that it was difficult to detect BLV in milk [14, 15]. For example, Kuckleburg et al. detected the BLV DNA in 82% and 59% of blood and milk samples, respectively, and the proviral loads in blood were significantly higher than those in milk [14]. The low proviral loads in milk might be explained by the presence of numerous components in milk (i.e., proteases, complex polysaccharides, lipids, and Ca^2+^ ions) that can interfere with DNA extraction and qPCR analysis, thereby, decreasing the detection sensitivity compared to that in the blood. Indeed, interference with PCR amplification was previously demonstrated when the limit of detection of BLV qPCR was assessed in the presence of the BLV provirus in a milk matrix [16]. Another explanation for the differences in proviral loads could be that less BLV-infected cells containing integrated BLV provirus are present in milk than in blood. Originally, data from Reber et al. indicated that fewer B-lymphocytes (the main target cells of BLV infection) were present in milk than in blood [43]. In this study, we purified genomic DNA of the same quality from milk and blood samples, and the expression of *BoLA*-*DRA* gene was found to be identical by PCR of the DNA extracted from the blood and milk samples (Figure 1). Therefore, the possibility that blood contains a larger number of BLV-infected lymphocytes than milk might be the most reasonable.

In this study, we provide the first evidence that milk cells from seven BLV-infected dam had BLV infectivity ex vivo. The EBL cow, A1, had a tumor in the posterior quarter of the mammary gland, a lymph node in the rear left breast, and a lymph node in the bilateral ilium (Figure 4). Furthermore, cow A1 showed a milk proviral load (6340 copies/10^5^ cells) higher than that in the blood from the same cow and other milk samples. Considering this information, the proviral load in milk appeared to be really high among the 12 milk samples tested and, therefore, it might be possible to detect the BLV infectivity of milk cells from one EBL cattle via the conventional LuSIA. However, whether the observed disease symptoms were related to the detection of BLV infectivity in milk remains unclear. We also successfully visualized the BLV-infected milk cells from six BLV-positive, but healthy, dams without lymphoma using a highly sensitive LuSIA optimized for milk cells. Collectively, the milk cells from these BLV-infected dams might include various somatic cells, such as leukocytes and epithelial cells. Previous studies suggest that BLV has a broad host range; therefore it can successfully infect various cell types in vitro and in vivo [44, 45]. Therefore, it is necessary to clarify which cell types of lymphocytes and various other cells in the milk were infected by BLV.

In this study, we modified a conventional LuSIA with CC81-GREMG cells by varying the number of CC81-GREMG cells, the number of milk cells, the co-culture period, and the stimulation by PWM. Decreasing the number of reporter cells seemed to increase the growth rate of reporter cells when compared with the conventional LuSIA. Furthermore, larger syncytia formed because the BLV-infected milk cells easily contacted uninfected cells and CC81-GREMG cells when the number of milk cells per well was increased. Finally, the addition of PWM clearly induced the formation of several large syncytia. PWM exerts mitogenic activity on B and T cells and induces cellular proliferation [46], which may have promoted the activation of milk cells and their contact with an optimal number of reporter cells. The improved LuSIA protocol based on CC81-GREMG cells was optimized using 1 × 10^4^ CC81-GREMG cells/well, 1 × 10^5^ milk cells/well, a 5-day co-culture period, and stimulation with 1 µg/mL of PWM (Table 3). It could also detect BLV infectivity in milk cells with higher resolution than was achieved in our previous protocol. More comprehensive studies are needed to confirm the mechanism of vertical BLV transmission through milk.

The present study successfully demonstrates that milk cells from BLV-infected dams have infectious ability ex vivo. Since present findings show the same tendency as previous study that lambs could be infected with BLV when inoculated with colostrum and milk which derived from BLV-infected cattle [13], we may conclude that milk is a risk factor for BLV vertical spread through cell to cell transmission. Nonetheless, few samples were evaluated, additional studies with more animals are required to further confirm these findings. In this study, we developed a strategy for estimating the BLV infectivity in milk, which should be quite useful for developing new methods with milk from BLV-infected cows to protect calves from BLV infection.

## Abbreviations

BLV: bovine leukemia virus
CoCoMo: coordination of common motifs
DMEM: Dulbecco’s modified Eagle’s medium
EBL: enzootic bovine leukemia
EDTA: ethylenediaminetetraacetic acid
EGFP: enhanced green fluorescent protein
ELISA: enzyme-linked immunosorbent assay
HIV: human immunodeficiency virus
LTR: long terminal repeat
LuSIA: luminescence syncytium induction assay
PCR: polymerase chain reaction
Ct: threshold cycle
PWM: pokeweed mitogen
WBC: white blood cell
FBS: fetal bovine serum
PBS: phosphate buffered saline
